# Identification of Reference Genes across Physiological States for qRT-PCR through Microarray Meta-Analysis

**DOI:** 10.1371/journal.pone.0017347

**Published:** 2011-02-24

**Authors:** Wei-Chung Cheng, Cheng-Wei Chang, Chaang-Ray Chen, Min-Lung Tsai, Wun-Yi Shu, Chia-Yang Li, Ian C. Hsu

**Affiliations:** 1 Department of Biomedical Engineering and Environmental Sciences, National Tsing Hua University, Hsinchu, Taiwan; 2 Institute of Athletics, National Taiwan Sport University, Taichung, Taiwan; 3 Institute of Statistics, National Tsing Hua University, Hsinchu, Taiwan; Centro de Investigación Príncipe Felipe, Spain

## Abstract

**Background:**

The accuracy of quantitative real-time PCR (qRT-PCR) is highly dependent on reliable reference gene(s). Some housekeeping genes which are commonly used for normalization are widely recognized as inappropriate in many experimental conditions. This study aimed to identify reference genes for clinical studies through microarray meta-analysis of human clinical samples.

**Methodology/Principal Findings:**

After uniform data preprocessing and data quality control, 4,804 Affymetrix HU-133A arrays performed by clinical samples were classified into four physiological states with 13 organ/tissue types. We identified a list of reference genes for each organ/tissue types which exhibited stable expression across physiological states. Furthermore, 102 genes identified as reference gene candidates in multiple organ/tissue types were selected for further analysis. These genes have been frequently identified as housekeeping genes in previous studies, and approximately 71% of them fall into Gene Expression (GO:0010467) category in Gene Ontology.

**Conclusions/Significance:**

Based on microarray meta-analysis of human clinical sample arrays, we identified sets of reference gene candidates for various organ/tissue types and then examined the functions of these genes. Additionally, we found that many of the reference genes are functionally related to transcription, RNA processing and translation. According to our results, researchers could select single or multiple reference gene(s) for normalization of qRT-PCR in clinical studies.

## Introduction

Reference genes (RGs) are widely used to normalize the expression level for removing potential artifacts caused by sample preparation and detection as well as to provide an accurate comparison of gene expression among different samples. Traditional reference genes (tRGs) are housekeeping genes (HKGs), such as ACTB, GAPDH, and HPRT, and usually serve as internal controls in Northern blot, RNase protection assays, conventional RT-PCR assays, and quantitative real-time PCR (qRT-PCR). The assumption is that these genes are defined as maintaining basic cellular functions [1] and are expressed at a constant level across samples, physiological states, and treatments. However, numerous studies have already shown that tRGs are regulated and their expression levels are varied under certain experimental conditions [2], [3], [4], [5].

qRT-PCR is often considered as the golden standard for quantitative gene expression analysis. However, the use of inappropriate RGs can result in incorrect findings if the expression levels of the chosen RGs are influenced by the experimental conditions [3], [6]. Researchers should make sure that the chosen RGs are suitable for the experiment they conducted. Thus, identification of RGs and their validation within specific biological conditions under investigation are critical issues.

Previous research identified RGs by selecting them from a list of tRGs for specified biological conditions according to the results of qRT-PCR [7], [8], [9], [10], [11], [12]. Microarray screening is an alternative approach and has the potential to identify novel RGs whose expression levels are more stable than that of tRGs. Moreover, the increasing amount of microarray data is an excellent source for the identification of genes with the most stable expression [13], [14], [15], [16]. Most research using microarray analysis identified RGs for specific biological conditions, for example, evolution [17], differentiation [18], development [19], treatment [20], cancer [13], [14], [21], [22], [23], [24], [25], [26], other diseases [27], [28], [29], [30] or comparing different physiological stages of a single organ [21], [23], [25]. A number of studies have identified RGs with relatively stable expressions across tissue types [31] and among metadata which pooled multitudes of arrays ignoring cell types and experimental conditions [32], [33]. However, no results have been reached for a consistent set of RGs. Many researchers assume that no RG is universally stable in its expression in all situations [14], [22], [23], [28], [34]. The ideal set of RGs depends on the biological conditions and should be selected and evaluated for each series of experiments.

This study aimed to identify RGs for clinical studies by meta-analysis of human clinical samples. These RGs had to demonstrate a stable expression across various physiological states in individual tissue/organ type. After the removal of poor quality arrays, 4,804 Affymetrix U133A arrays performed on human clinical samples were selected from the M^2^DB, a microarray meta-analysis database [35]. These arrays were classified into 4 physiological states and 13 organ/tissue types. Genes showing stable expressions within and between physiological stages for a single tissue were identified as RGs for that particular tissue. Our results recommended a number of sets of RGs for various organ/tissue types. Additionally, we have found that the genes that are frequently identified as RGs for multiple organ/tissue types are highly related to the functional category, Gene Expression (GO: 0010467). These genes are frequently classified as HKGs in previous studies. Besides, our results suggest that RGs identified in this study are candidates as control genes for qRT-PCR in clinical studies.

## Analysis

### Microarray data collection, quality control, and pre-processing

Expression data were collected from the M^2^DB, which compiles more than 10,000 well-annotated, published, human clinical Affymetrix GeneChip arrays. We excluded poor quality arrays (8% of the total), that did not match the criteria of the 95 percentile of PMVO [36], according to the QC metrics of the M^2^DB. Then, according to the annotation of the M^2^DB, samples related to the same organ/tissue type and the same physiological state were classified into a single group. An organ/tissue type was included into this study if it has at least two groups, which contained at least 10 HG-U133A arrays, in the organ/tissue type. In summary, this study included 4,804 HG-U133A arrays classified into 13 organ/tissue types and 4 physiological states (Normal, Abnormality, Disease, and Cancer or Tumor). Table 1 gives the summary of the number of arrays classified in each organ/tissue and physiological state. The data uniformly processed by the GC Robust Multi-array Average (GCRMA) algorithm [37] were downloaded from the M^2^DB. Intensities (without log transformation) of the probe sets with the same Entrez GeneID were averaged to represent the expression of the corresponding gene.

**Table 1:** Summary of arrays classified into 4 physiological states and 13 organ/tissue types.

Organ/Tissue Types	Physiological States				
	Normal	Cancer or Tumor	Disease	Abnormality	Total
blood	252	403	514	137	1,306
lung	44	92	66	128	330
bone marrow	39	559	19	0	617
brain	229	139	202	0	570
uterus	18	15	13	0	46
breast	10	1,229	0	3	1,242
kidney	9	21	0	10	40
bladder	12	64	0	0	76
lymph node	12	33	0	0	45
prostate	15	59	0	0	74
testis	17	102	0	0	119
muscle	75	0	109	44	228
heart	30	0	81	0	111
Total	762	2,716	1,004	322	4,804

### Selection of Reference Gene Candidates

The definition of an RG in this study is that a gene stably expressed for each organ across different physiological states. RGs for each organ/tissue type were identified using the following criteria:

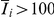
 and FP>80%.

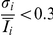

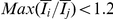

Where 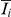 and 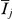 denote the mean intensity of the gene in arrays of ith and jth physiological states respectively. 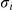 is the standard deviation of intensity in ith physiological states. Max() is the maximum ratio of mean intensity. For a gene, FP is fraction Present which is the fraction of arrays called present in a single organ/tissue type [38]. The first criterion identified genes that are truly expressed in a tissue. For each gene, the expression values were averaged for each physiological state. A gene was retained if the average expression level exceeded the selected threshold value 100 and FP was larger than 80%. Filtering data by FP increases the correlation between Affymetrix GeneChip and qRT-PCR expression measurements [39]. Genes with their expression values satisfy these two thresholds are most likely to be truly expressed. The second criterion used the coefficient of variation, standard deviation divided by mean intensity, to verify whether the genes exhibited stable expressions in a physiological state. The third criterion used fold change of expression to filter out genes that differentially expressed across physiological states in a single organ/tissue type. The fold change refers to the ratio of mean intensity of physiological states and represents the expression differences between physiological states. Table 2 shows the number of genes which are stably expressed within individual physiological state (the first and second criteria), stably expressed across physiological states (the first and third criteria), and qualified as RGs (all three criteria) for each organ/tissue type. For example, by apply the first two criteria, the counts of genes stably expressed within the four physiological states in blood are 133, 203, 479, and 238, respectively. By applying the first and third criteria, there were 162 genes stably expressed across physiological states in blood. Finally, 11 genes passed all three criteria were identified as RGs for blood. Data S1 gives the complete lists of RGs for respective organ/tissue types.

**Table 2:** Summary of the number of genes passed different criteria in 13 organ/tissue types.

Organ/Tissue types	Stable Within Physiological States*				Stable Between Physiological States§	RGs¥
	Normal	Cancer or Tumor	Disease	Abnormality		
blood	133	203	479	238	162	11
lung	211	117	768	581	195	16
bone marrow	186	66	382	-	301	21
brain	184	60	491	-	657	15
uterus	761	1,454	1,548	-	1,271	276
breast	352	108	-	2,263	378	17
kidney	201	1,041	-	2,542	421	31
bladder	362	200	-	-	1,385	89
lymph node	2,212	495	-	-	1,030	150
prostate	734	106	-	-	1,989	65
testis	238	173	-	-	713	13
muscle	327	-	198	478	1,103	93
heart	742	-	794	-	2,406	250

*The criterion is that the CV of the intensity of the gene in the physiological state is smaller than 30%. CV, coefficient of variation, is equal to standard deviation divided by mean.

§ The criterion is that the maximum of the fold change of mean intensity between physiological states is smaller than 1.2.

¥ The number of genes stably expressed within and between physiological states.

### Frequent Reference Genes

**Figure 1:**
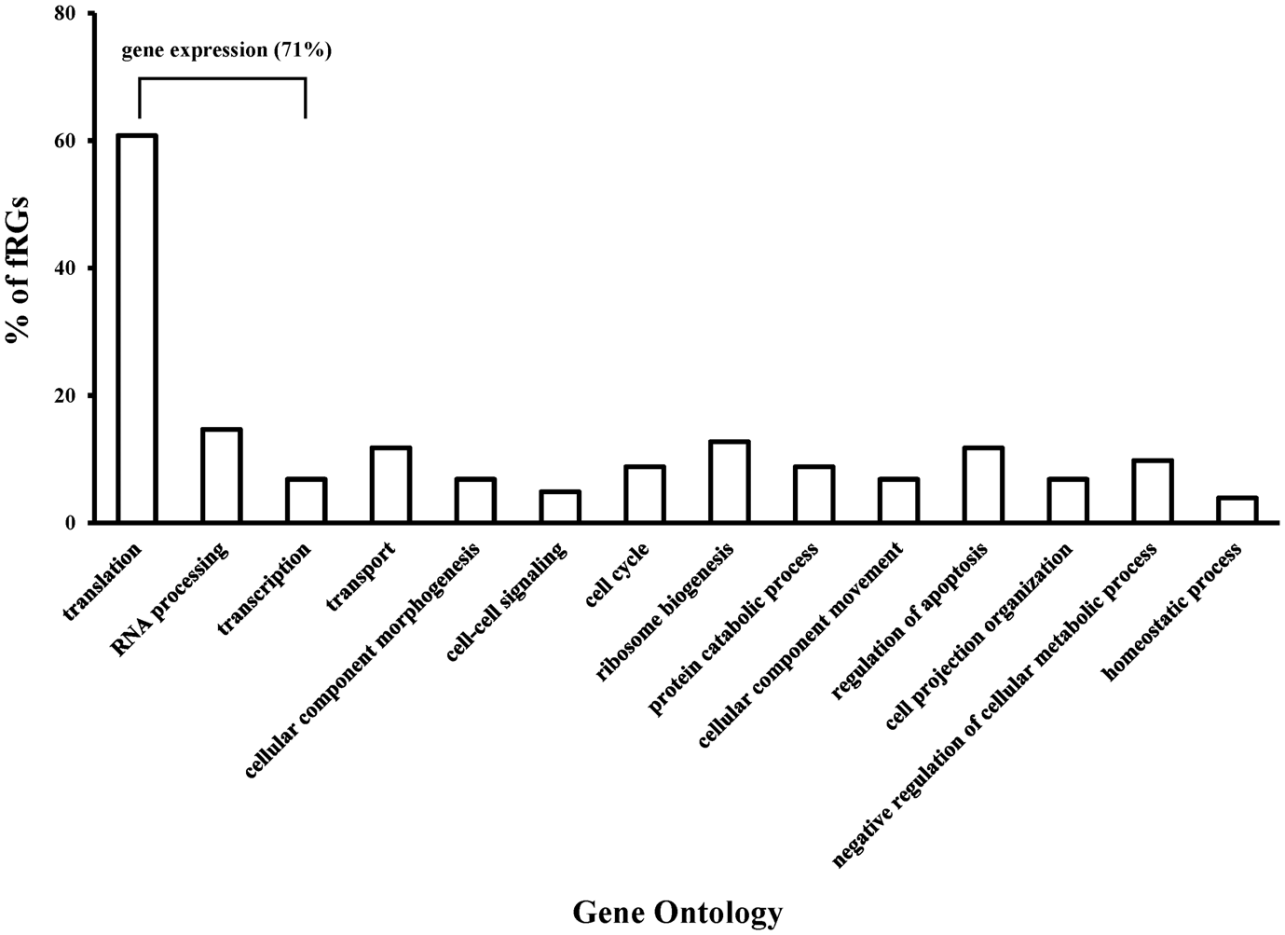
Gene Ontology Functional analysis of fRGs. The percentage of fRGs counted in 14 GO terms which give a broad overview of gene function. Gene expression is the parent term of transcription, translation, and RNA processing in Gene Ontology and contains 71% of fRGs.

The genes which were identified as RGs for at least three organ/tissue types are denoted as frequent reference genes (fRGs). Table 3 displays a list of 102 fRGs and the corresponding numbers of organ/tissue types for which the RGs were identified. Some tRGs, such as ACTB, B2M, UBC, RPL13A and RPLP0, are also on this list. Gene ontology was used to analyze the gene function of fRGs. A set of GO terms (14 terms) was chosen to give a broad overview of gene function. Figure S1 generated by QuickGO [40] is a graphical view of the term lineage of these 14 terms in Gene Ontology. Figure 1 shows the percentage of fRGs counts in these 14 terms. Approximately 61%, 15%, and 7% of fRGs belong to Translation (GO: 0006412), RNA Processing (GO: 0006396), and Transcription (GO: 0006350) respectively. Moreover, these three terms are children of Gene Expression (GO: 0010467) (Figure S1). Approximately 71% of the fRGs fall into this functional category. These are basic cellular functions referring to HKGs. When compared with 8 lists of HKGs identified by microarray or EST analysis in 7 previous studies [16], [41], [42], [43], [44], [45], [46], fRGs were frequently classified as HKGs in these lists. Furthermore, the percentages of these HKGs lists falling into Gene Expression (GO: 0010467) range from 22.4 to 35.1 (Table 4). These percentages are much lower than that of fRGs. In addition, these 14 terms cover 84% of fRGs. The other 16% of fRGs do not belong to these 14 GO terms, and half of these genes do not refer to any GO terms.

**Table 3:** fRGs and the corresponding numbers of organ/tissue types for which fRGs were identified.

Num. of organ/tissue types	Gene Symbol
3	SEPT2, ATG4B, B2M, BTF3, DAZAP2, DDB1, DDX17, EIF4G2, ENSA, EWSR1, FNTA, HDAC3, HDLBP, HMGB1, HMGN2, HNRNPA1, MORF4L1, MTCH1, PI4KB, PUM1, RPL10, RPL11, RPL17, RPL19, RPL22, RPL4, RPL5, RPS12, RPS15, RPS28, RPS3, RPS7, TBC1D9B, TCEB3
4	ACTB, CCDC72, EEF1G, EEF2, FTHP1, GDI1, GTF2F1, GTPBP6, RPL12, RPL24, RPL27A, RPL30, RPL37, RPL38, RPL39, RPL7, RPL7A, RPLP0, RPLP1, RPS16, RPS2, RPS24, RPS25, RPS27A, RPS3A, RPS4X, RPSA, SKP1, SNRPB2, SRP14, USP34
5	ACTG1, EEF1D, EIF1, MYL12B, OAZ1, RPL13A, RPL15, RPL21, RPL27, RPL31, RPL32, RPS13, RPS14, RPS15A, TOX4, UBA52
6	PNN, RPL34, RPL9, RPS10, RPS11, RPS17, RPS18, RPS23, RPS27, RPS29, UBB, UBC
7	NACAP1, RPL23A
8	EEF1A1, LRRC40, RPS20
9	RPL41
10	RPL37A, TPT1
11	HUWE1

**Table 4:** Comparison of fRGs with HKG lists of previous studies.

References	Tech.	% of overlap*	% in Gene Expression (GO: 0010467)§
Warrington et al. 2000 [16]	Microarray	59.8	31.9
Hsiao et al. 2001 [46]	Microarray	58.8	35.1
Eisenberg et al. 2003 [45]	Microarray	43.1	27.2
Tu et al. 2006 [44]	Microarray	75.5	22.4
Zhu et al. 2008 [42]	EST	92.2	24.5
Zhu et al. 2008 [42]	Microarray	85.3	26.1
Dezso et al. 2008 [43]	Microarray	81.4	24.3
She et al. 2009 [41]	Microarray	68.6	29.1
fRGs	Microarray	-	70.6.

*The percentage of fRGs falls into HKG lists.

§ The percentage of genes in these lists falls into Gene Expression (GO: 0010467) category in Gene Ontology.

### Expression profiles of tRGs and fRGs

**Figure 2:**
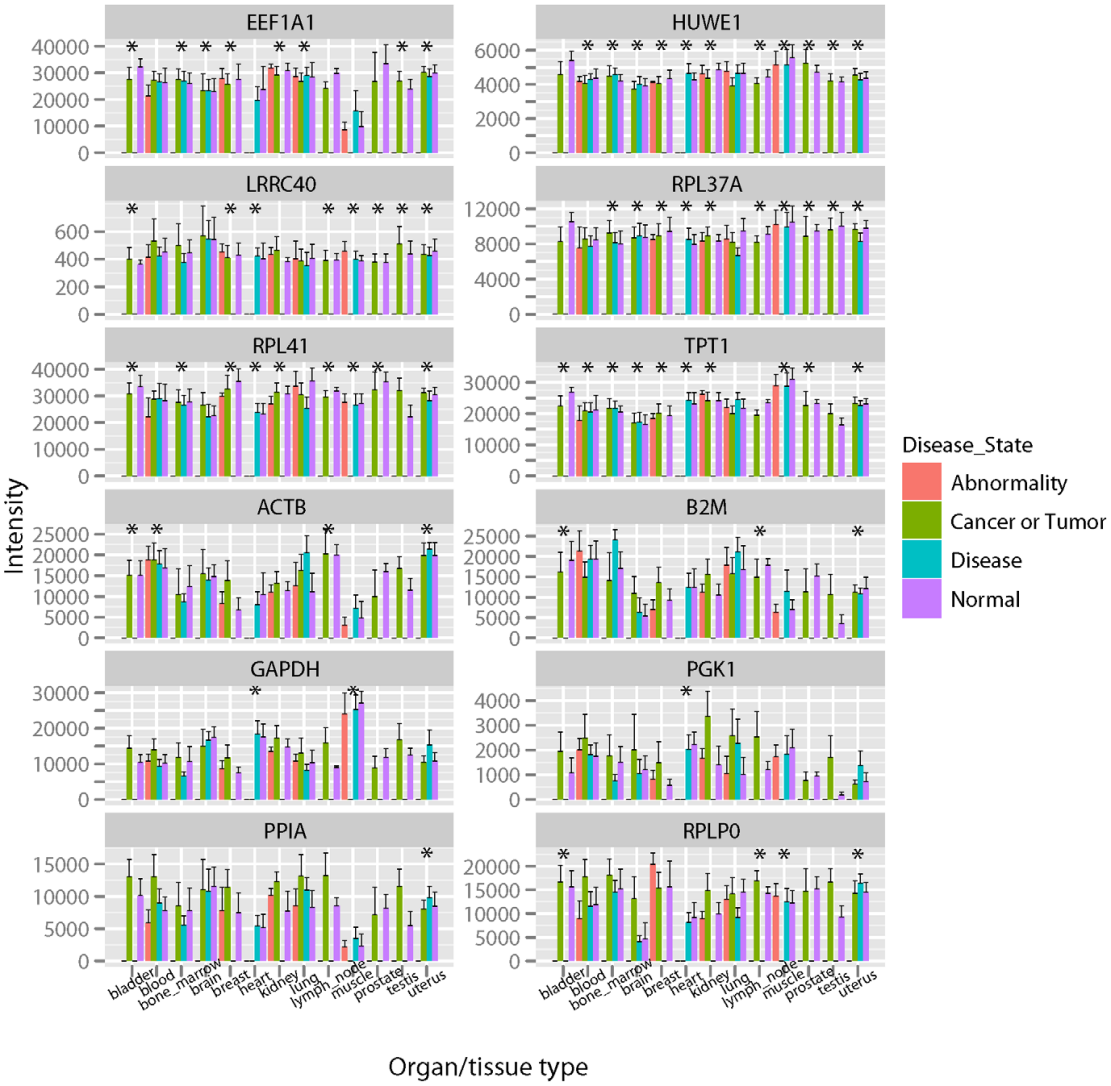
Expression profiles of 6 tRGs and 6 fRGs for 4 physiological states in 13 organ/tissue types. The upper and lower halfs of the figure are 6 fRGs and 6 tRGs respectively. The error bar is the standard deviation of intensity. * denotes the gene identified as RG in the organ/tissue type.

Six tRGs and six fRGs were selected to examine the expression profiles. The 6 housekeeping genes (ACTB, B2M, GAPDH, PKG1, RPLP0, and PPIA) have been commonly used as reference genes for qRT-PCR in numerous studies. In this study, the 6 fRGs (HUWE1, TPT1, EEF1A1, LRRC40, RPS20, RPL37A, and RPL41) are the most frequently identified RGs in various organ/tissue types (Table 3). Three of the housekeeping genes, ACTB, B2M, and RPLP0, are also identified as fRGs. Although the other three housekeeping gene are not fRGs, they are still identified as RGs for one or two organs/tissue types. Figure 2 depicts the intensity profile of the 12 genes (6 tRGs and 6 fRGs) in various physiological states of 13 organ/tissue types. The RGs exhibit consistent expressions in the corresponding organ/tissue type. The 6 fRGs exhibit more stable expression than the 6 tRGs do both within and between organ/tissue types.

## Discussion

We examined the variability of gene expression within and between various physiological states in 13 organ/tissue types. Lists of RGs were identified for the corresponding organ/tissue types. Clinical research usually focused on various physiological states for a single organ/tissue type (such as cancer classification [47], [48], [49]). The relative expression level of an ideal RG for clinical studies should not be significantly influenced by physiological states. Previous studies, which used microarray screening to identify RGs, mostly focused on a specific physiological state in an organ/tissue type. Some research identified universal RGs by pooling all of microarray data from public repositories ignoring organ/tissue types and physiological states [31], [32], [33]. Different from them, our study broadly searched RGs in various physiological stages of 13 organ/tissue types. To achieve this goal, we classified samples into four physiological states according to information found in the M^2^DB. Then, we applied several criteria to identify expressed genes with consistent expression within and between physiological states as RGs. Genes satisfied these selection criteria indeed exhibited stable expression and results indicated that the tRGs are not always the best choice for reference of qRT-PCR (Figure 2). Although numbers of genes in our RG list had been reported as RGs for some experimental conditions in previous studies, our results specified which gene could be RG in particular organ/tissue types. For example, ACTB, the most frequently used tRG, is also in our fRGs list, but we suggested that ACTB can only be served as RG in three organ/tissue types out of the total thirteen organ/tissue types which we investigated. Furthermore, unlike some previous studies, our results indicated that there is no universal RG for all experimental conditions listed in our study. As the result, it also shows that choosing randomly any HKGs for normalization is risky and may lead to erroneous results.

With rapidly accumulating metadata, microarray meta-analysis is becoming more important in microarray research. One major concern is that as more datasets are included into analysis, the more variance could contribute to the result. Ramasamy et al. had suggested several key issues for microarray meta-analysis [50]. Using pre-processed data based on different algorithms will introduce variations into meta-analysis and the resulting data are unlikely to be directly comparable. As Ramasamy et al. point out, even for studies conducted using the same microarray platform; the raw data should be uniformly pre-processed and normalized using the same algorithm to remove systematic biases for all tested datasets. Several studies have suggested considering data quality within the context of microarray meta-analysis [50], [51], [52]. Poor quality data must be identified and eliminated during data processing [50], [53]. In this study, we adopted single platform for analysis to avoid the variance of combining different platforms, and then uniformly pre-processed all arrays to eliminate the technical variance of data transformation and removed poor quality arrays to alleviate laboratory-to-laboratory variance [35]. Moreover, we used the 12 tRGs and fRGs in Figure 2 to evaluate the effect of QC (Figure S2). The CV of intensity for these genes with QC was lower compared to those without QC. This result suggests that including poor quality arrays could lead to increase expression variation. The advantageous effect of excluding poor quality data is apparent when processing muscle tissues. More than 40% of muscle sample arrays were identified as poor quality arrays (8% of total arrays are poor quality). This result shows that expression variations of RGs are greatly reduced when poor quality muscle arrays were excluded.

Most genes included in lists of fRGs were commonly referred to as HKGs in previous studies (Table 4). To a certain extent, this result is in line with the original concept of using HKGs as RGs for normalization. However, contrary to commonly held assumptions, no HKGs were consistently expressed across all tissues in our study. Moreover, no genes maintained a stable expression level under all conditions (various organ/tissue types and physiological states) (Table 4). In fact, this observation has been mentioned in previous studies [14], [22], [23], [28], [34] which presumed there is no universal RGs for all experimental conditions. Furthermore, approximately 71% of fRGs' were related to the function of Gene Expression (including Transcription, Translation, and RNA Processing). The percentage is much higher than those of HKGs lists by previous studies (Table 4). Consequently, fRGs are highly related to HKGs and maintained at relatively stable level. This result indicates that the genes in the Gene Expression (GO: 0010467) category are more likely to be stably expressed across physiological states and organ/tissue types. This may imply these genes play more important roles than general HKGs. Besides, we found that half of the fRGs were ribosomal protein genes. A meta-analysis study conducted by de Jonge et al. revealed 15 reference genes with the most constant expression, and 13 out of 15 genes were ribosomal proteins [32]. In contrast, Thorrez et al. demonstrated that ribosomal protein genes exhibited important tissue-dependent variations in mRNA expression [54]. Thorrez's results were based on the study of 70 microarrays, representing 22 tissues. The authors cautioned against using ribosomal protein genes as a reference [54]. Our study, which preserved more sample conditions, resolves the contradictory conclusions by these two studies. Our results depicts that some ribosomal protein genes maintained relative stability of expression across organ/tissue types, however, some ribosomal proteins exhibited significant tissue-dependent expression (for example, RPLP0 in Figure 2). The RGs identified in this study expressed stably across physiological states in a single organ/tissue type. Thus, a number of ribosomal protein genes tallied with the criterion could be identified as RGs. For example, in this study, more than half of RGs for breast are ribosomal protein genes, which is consistent with the results of a meta-analysis to identify RGs for breast cancer [26]. However, if the experiment is conducted by various organ/tissue types, it required further verification to use ribosomal protein genes as reference.

UBB, UBC, and UBA52 in the list of fRGs are known as functions related to protein ubiquitination, as well as numerous essential cellular functions. They have been identified as RGs in breast cancer [26]. UBC is a tRG and has also been identified as an RG in colon cancer [14]. TPT1 was initially described as a growth-related protein, and it was recently shown being involved in calcium homeostasis [55]. This implies the expression stability of TPT1 could influence the calcium stability in cells. It could be the reason that TPT1 was identified as RG in previous studies [14], [29] and for 10 organ/tissue types in this study. RPL41 and EEFA1 in the list of fRGs have also been recognized as RG for liver [23] and myocardium [29] respectively. GAPDH, the most common tRG, was identified as RG only for heart and muscle in this study, but this is partially consistent with the previous study which identified GAPDH as a RG for myocardium [29]. HUWE1, which is related to histone ubiquitination [56] and protein polyubiquitination [57], was the top-ranked RG in our result. Although HUWE1 was not the most stable gene in individual organ/tissue type, it was the gene most frequently identified as RG in this study, and suggested to be a novel RG candidate for clinical studies.

Geometric averaging of multiple RGs rather than using single RG for normalization of qRT-PCR is an alternative strategy [58]. We have supplied lists of RG candidates for researchers to confirm their qRT-PCR results under particular experimental conditions. Choosing several RG candidates from our RG lists to perform qRT-PCR could help researchers to confirm one or multiple RGs for use as references.

For some organ/tissue types, there were only dozens of samples for identifying RGs, despite the thousands of arrays included in this study (Table 1). This might underestimate the variance of expression among individuals or physiological conditions and might lead to increased false positive rate. For example, 276 RG candidates were identified for the uterus (Table 2). There is a limitation of accuracy in identifying RG upon small number of samples. However, our RG list can be good candidates for researchers to identify the true RG by qRT-PCR but not choosing HKGs randomly as reference. Researchers can exclude unsuitable RGs which had been shown variable expression in our results. Using the same example, the most used tRG, GAPDH, is not included in the 276 RG candidates for the uterus. Thus, researchers could choose several candidate genes in our list for further validation by qRT-PCR but GAPDH. In the future, with rapidly accumulated microarray metadata, we could gather more clinical arrays and subdivide them by detailed physiological states and organ/tissue types. Accordingly, the more accurate RGs could be identified for clinical studies.

In summary, this study performed microarray meta-analysis to compile lists of RG candidate for 13 organ/tissue types. We provided lists of RG candidates for researchers to select single or multiple genes as references for the normalization of qRT-PCR in clinical studies. We also found that fRGs were recognized as HKGs in previously studies and about 71% of fRGs were functional annotated to Gene Expression (GO:0010467). The percentage is also much higher than that of HKG lists. To our best knowledge, this is the first study considering different physiological states as well as identifying RGs for various organ/tissue types. In our results, the tRGs are not the best choice for reference of qRT-PCR in most conditions, and the RGs identified in this study are more reliable than tRGs for normalization in qRT-PCR for clinical studies.

## Supporting Information

Data S1 The complete lists of RGs for the 13 organ/tissue types. For each gene, the CV and mean intensity of various physiological states are also included in this file.
(XLS)

Figure S1 The lineage of 14 GO terms.
(TIF)

Figure S2 The CV of intensity of 12 genes in 13 organ/tissue types with/without QC filitering.
(TIF)
