# Supplementary figures and images for: Identification of Reference Genes across Physiological States for qRT-PCR through Microarray Meta-Analysis
Source: PLoS One. 2011 Feb 24;6(2):e17347. doi: 10.1371/journal.pone.0017347 (PMC3044736; doi:10.1371/journal.pone.0017347)

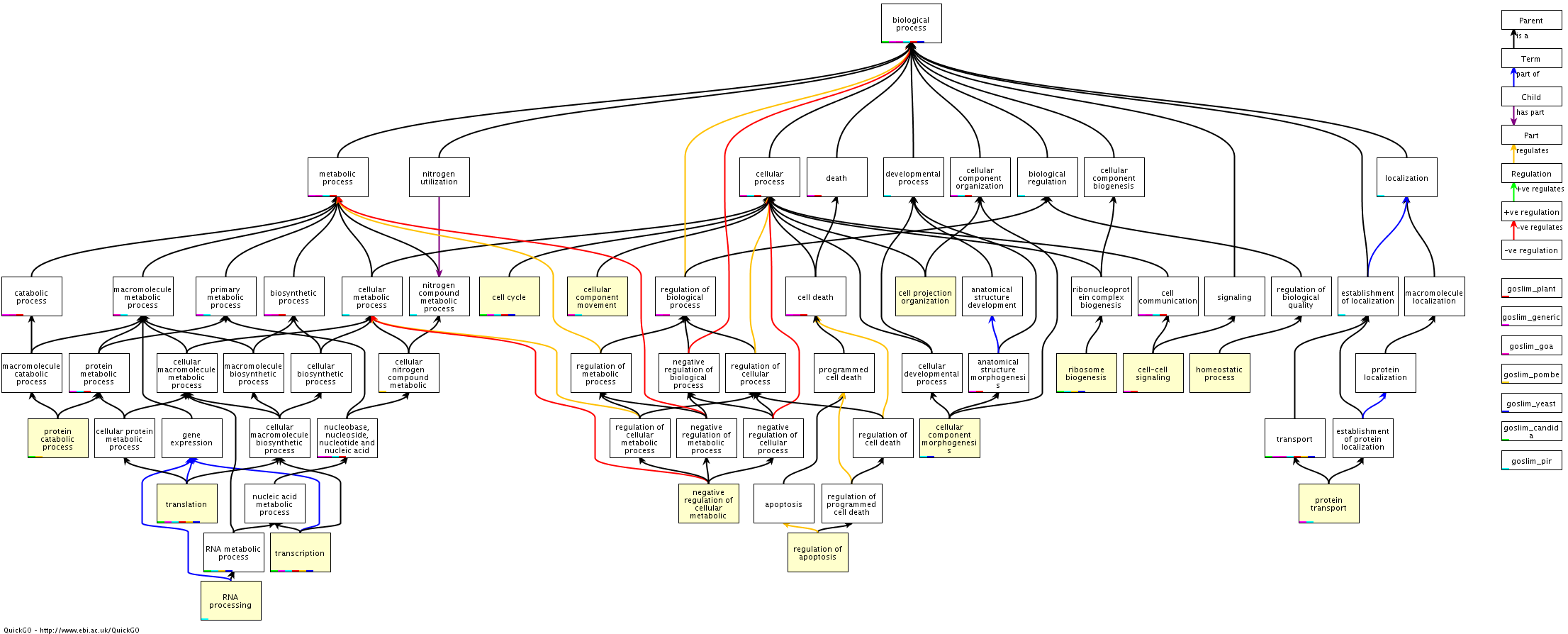

Supplement: Figure S1 — The lineage of 14 GO terms. (TIF) [file pone.0017347.s002.tif]

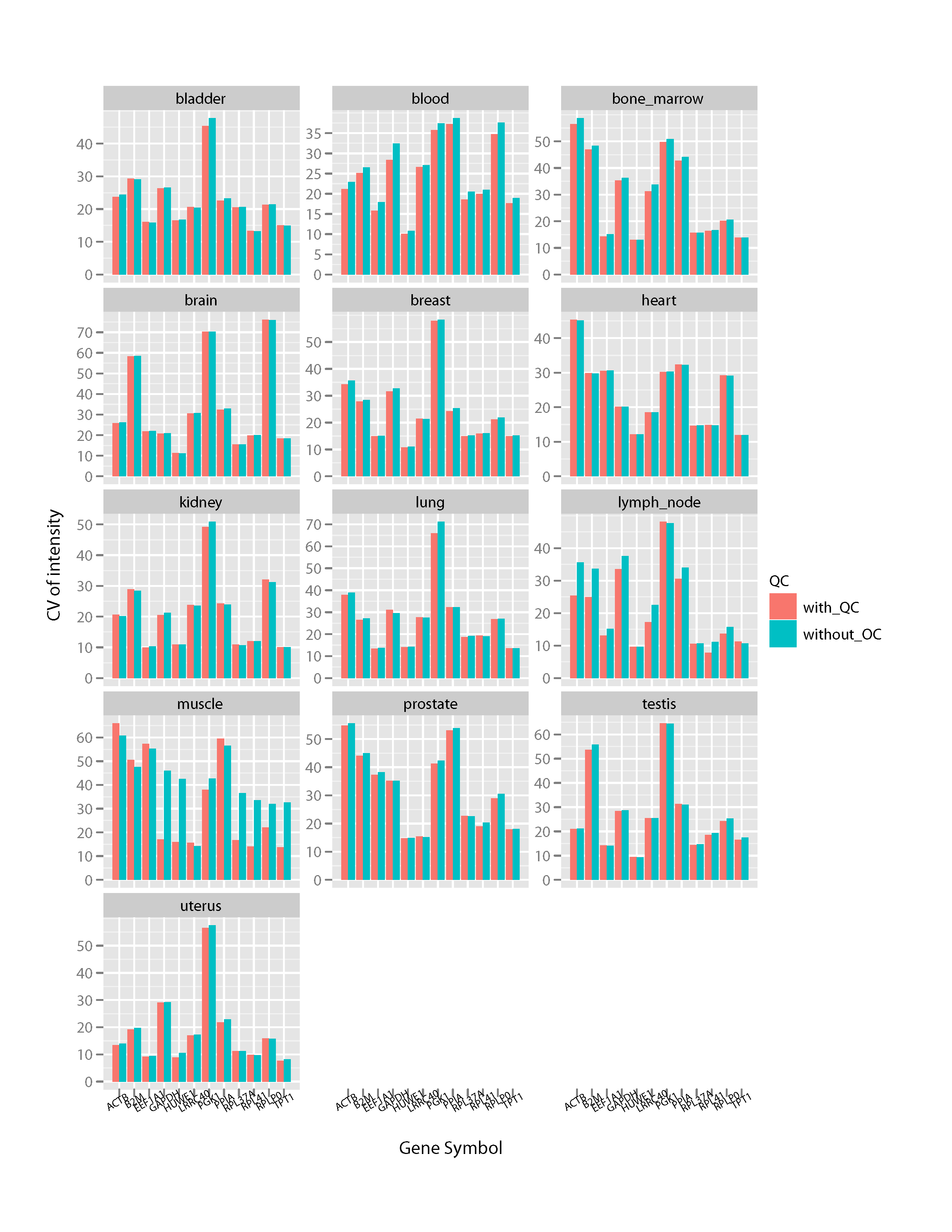

Supplement: Figure S2 — The CV of intensity of 12 genes in 13 organ/tissue types with/without QC filitering. (TIF) [file pone.0017347.s003.tif]
